# Effects of Atorvastatin on Oxidative Stress Biomarkers and Mitochondrial Morphofunctionality in Hyperfibrinogenemia-Induced Atherogenesis

**DOI:** 10.1155/2014/947258

**Published:** 2014-10-22

**Authors:** María de la Paz Scribano, María del Carmen Baez, Becerra Florencia, Mariana Denise Tarán, Signorini Franco, Ariel G. Balceda, Mónica Moya

**Affiliations:** ^1^Cátedra de Física Biomédica, Facultad de Ciencias Médicas, Universidad Nacional de Córdoba, X5000 Córdoba, Argentina; ^2^Instituto de Investigación en Ciencias de la Salud Humana (IICSHUM), Universidad Nacional de La Rioja, La Rioja, Argentina; ^3^Becaria Secyt, Universidad Nacional de Córdoba, Córdoba, Argentina; ^4^Cátedra de Física Biomédica, Facultad de Ciencias Médicas, Universidad Nacional de La Rioja, F5300 La Rioja, Argentina

## Abstract

Relationship between hyperfibrinogenemia (HF), oxidative stress, and atherogenesis was established. Effect of atorvastatin (Ator) was assessed. Wistar male (6 months) rats were studied: Ctr, control, without HF induction; Ctr-Ator, without HF treated with atorvastatin; AI, atherogenesis induced, and AI-Ator, atherogenesis induced and treated with atorvastatin. Atherogenesis was induced by daily adrenaline injection (0.1 mL/day/rat) for 90 days; treatment started 15 days after induction. Fibrinogen (mg/dL) and nitric oxide (NO) were measured in plasma (mM) and superoxide dismutase (SOD) (U/mL) in red cell lysate by spectrophotometry. Slices of aorta were analyzed by electron microscopy (EM). ANOVA and chi-square test were used; *P* < 0.05 was established. There were no significant differences between Ctr and Ctr-Atorv in fibrinogen, NO, and SOD values. Comparing Ctr with AI an increase of fibrinogen is observed (*P* < 0.001), but it decreased after administration of atorvastatin in AI-Ator (*P* < 0.001). NO diminished in AI relative to Ctr and increased in AI-Ator (*P* < 0.001). SOD showed an increase in AI and AI-Ator compared to Ctr (*P* < 0.001). EM revealed expansion of intermembrane space and disorganization of crests in AI. In AI-Ator mitochondrial areas and diameters were similar to control. Atorvastatin normalizes HF, stabilizes NO, increases SOD, and produces a partial regression of mitochondrial lesions.

## 1. Introduction

Cardiovascular diseases represent one of the main causes of morbimortality in developed and emerging countries. Atherogenesis has been established as the pathophysiological substrate of these pathologies, leading to abundant research on atherogenic triggers, progression, and possible treatments, as well as increased primary and secondary preventive measures. However, a high incidence of acute cardiovascular events has been reported in subjects classified as healthy according to the Framingham stratification criteria or to the guidelines elaborated by the National Cholesterol Education Program—Adult Treatment Panel III (ATP III) [1]. New risk factors and vascular disease markers, such as hyperfibrinogenemia (HF), participate in platelet aggregation, modulate endothelial function, promote the proliferation of smooth muscle, and express the inflammatory component in atherogenesis [2–4], mediated by TNF-*α* which reflects the endothelial activation level. Some years ago, several studies proved that fibrinogen values behave as risk indicators of an adverse cardiovascular event due to their participation in the stages of subclinical atherosclerosis [5, 6]. In previous studies, we have demonstrated that experimentally induced HF generates atherogenic lesions in the thoracic aorta of rats compatible with Stary's classification modified by Fuster [7]. These anatomopathological changes are generated by endothelial chemical activation and stimulate proinflammatory and prooxidative activity, which is reflected by oxidative stress with variations in nitric oxide (NO), a highly reactive and lipophilic molecule associated with reactive oxygen species (ROS) that easily spreads through membranes without the need for active receptors [8]. Many studies support the hypothesis that the formation of ROS in different cell compartments contributes to the oxidative modification in the arterial wall, altering the intracellular reduction-oxidation homeostasis [9]. When oxidative stress is caused, high and persistent fibrinogen concentrations induce the pathophysiological effects of NO, binding to the superoxide anion (O^2−^) at very high speeds in the dysfunctional control limit, resulting in the formation of peroxynitrite (ONOO^−^). This is the pathway that supposedly initiates endothelial homeostatic imbalance, generating atherogenesis during states of hyperfibrinogenemia [9]; the uncontrolled production of ROS apparently has an impact at the cellular level and induces lipid peroxidation in the cell membrane, spreading its effects to the mitochondrial level [10]. Mitochondria supply most of the energy needed for cellular activity and constitute the main source of free radicals at the level of the electron transport chain located in the inner mitochondrial membrane [11]. Control of the mitochondrial function depends on the concentration of NO and the oxygen level; they act on concentration and gradient intervals and there are critical points in which both variables are intercepted and produce peroxynitrites. Due to the location of the respiratory chain, it is inevitably damaged by the superoxide ion which leads to an increase in oxygen free radicals, generating a vicious cycle of deterioration of the mitochondrial function and morphology [12, 13]. If the process perpetuates, this response is indefinitely amplified; on the contrary, if the atherogenic stimulus disappears or the inflammatory activity and oxidative stress are controlled, there could be a regression of lesions that regenerates the endothelial function and structure [14, 15]. There is a mechanism that physiologically protects cells from oxidative stress and its cardiovascular consequences: a group of enzymes called endogenous antioxidants, which are subject to genetic and metabolic regulation and protect against the formation of new free radicals [16, 17]. Superoxide dismutase (SOD) is one of these enzymes and its function is to eliminate superoxide ion by H_2_O_2_ and O_2_ dismutation, avoiding the reaction with susceptible biological molecules [16–18]; however, relatively high levels of SOD in the arterial interstitium may not be enough to prevent the formation of peroxynitrites or other nitrate species. On the other hand, there are exogenous antioxidant drugs such as atorvastatin, a 3-hydroxy-3-methylglutaryl-CoA (HMG-CoA) reductase inhibitor, which, besides being a hypolipemiant agent, has pleiotropic effects that are independent of the competitive and reversible inhibition of HMG-CoA reductase. These effects include antioxidant, anti-inflammatory, and antithrombotic actions, a decrease in the cytokine production, and an increase in the production and availability of nitric oxide [15]. According to the aforementioned and considering that atherogenesis is a complex process characterized by a combination of factors related to inflammatory phenomena and lipid accumulation, atorvastatin, whose action mechanisms affect both components, is a good therapeutic strategy for this pathology [19].

The purpose of this study was to determine, in an experimental model of HF-induced atherogenesis, if atorvastatin normalizes hyperfibrinogenemia and the bioavailability of NO, as well as SOD activity, and to analyze the likely reversal of the mitochondrial morphofunctional alterations in aortic layers.

## 2. Materials and Methods

Male Wistar adults rats with an average weight of 280 ± 20 g were used. Animals were bred and housed under controlled conditions, maintained at room temperature (20°C ± 2°C), and fed on a balanced Cargill's diet. The studies were carried out according to the Guide for the Care and Use of Laboratory Animals published by the US National Institute of Health (NIH publication number 85-23, revised 1996) and was approved by the ethical commission of the School of Medicine of the National University of Córdoba (resoluteness 2/2012). A total of 48 rats were used and each group had 12 animals which were sequentially studied and classified into the following experimental situations:

- Ctr group: control (without hyperfibrinogenemia induction) (A),
- Ctr-Ator: control (without hyperfibrinogenemia induction) + treatment with atorvastatin for 75 days (B),
- AI group: hyperfibrinogenemia induced for 90 days (C),
- AI-Ator group: hyperfibrinogenemia induced for 90 days + treatment with atorvastatin for 75 days (D).

All animals survived and no animals were excluded in any of the groups studied.

Hyperfibrinogenemia was induced via subcutaneous injections of adrenaline in the dorsum of the animal (0.1 mL/day/rat) for 90 days. Atorvastatin (Torivas) was administered PO with a 1 mL syringe fitted with a catheter on one end, allowing the adequate dose to be deposited on the esophagus and hence avoid regurgitation by the animal; the dose was 0.035 mg/day/rat and it began on day 15 after the first HF induction and for a period of 75 days. Blood was obtained by decapitation of the animals that had previously been anesthetized with Ketalar (10 mg/Kg/animal) 72 hours after the last injury, coinciding with the 90-day induction period. The blood was collected in Petri dishes with an anticoagulant mix made up of ammonium and potassium oxalate in a 2 : 1 ratio. For SOD, EDTA was used as anticoagulant and centrifuged at 3000 rpm for 15 minutes to obtain plasma and red cell lysate. Material for the EM analysis was obtained by “*in toto*” slices of 2 mm rings of thoracic aorta for each group studied. The tissue was fixed for a period of at least 2 hours at room temperature in Karnovsky's fixative [20] made up of a mix of 4% formaldehyde and 1.5% glutaraldehyde in 0.1 M cacodylate buffer. Ultrathin sections were obtained with a diamond knife on a JEOL-JUM-7 ultramicrotome, mounted on nickel grids, stained with uranyl acetate in alcohol solution followed by lead citrate, and observed and photographed with a Leo 906E (Carl Zeiss, Jena, Germany) electron microscope.

Plasmatic fibrinogen (mg/dL) was measured by the Ratnoff and Menzie method [21]. Besides, plasma NO (*μ*M) measurement was carried out with Griess reaction [22], SOD activity (U/mL) was assayed in red cell lysates using a Randox Kit [23], and all were determined by spectrophotometry (Metrolab, Buenos Aires, Argentina).

### 2.1. Statistical Analysis

The results of independent variables (animal groups studied) in relation to the complexes were analyzed using* Infostat* (InfoStat version 2008, InfoStat Group, Argentina). Normality and homogeneity of variances were determined using the Shapiro-Wilk and Levene's test. The difference between the groups was analyzed with a MANOVA and compared post hoc with Hotelling's *T* ^2^ test. As regards mitochondrial morphology, the program Axiovision 4.8 (2009, Carl Zeiss, Jena, Germany) was used to evaluate the mitochondrial structure and Fisher's exact test was used to compare categorical variables. A significance level of *P* < 0.05 was established for all measures.

## 3. Results

**Figure 1:**
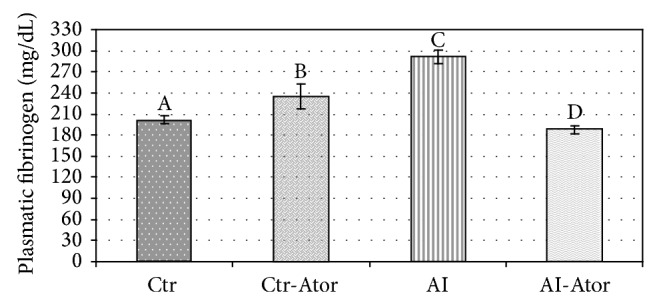
Plasmatic fibrinogen values in rats with induced HF treated with atorvastatin (*n* = 12). Crt versus Ctr.Ator ND; Ctr versus AI *P* < 0.0001; Ctr versus AI-Ator *P* < 0.001; Ctr-Ator versus AI *P* < 0.001; Ctr-Ator versus AI-Ator *P* < 0.0001; AI versus AI-Ator *P* < 0.001.

**Figure 2:**
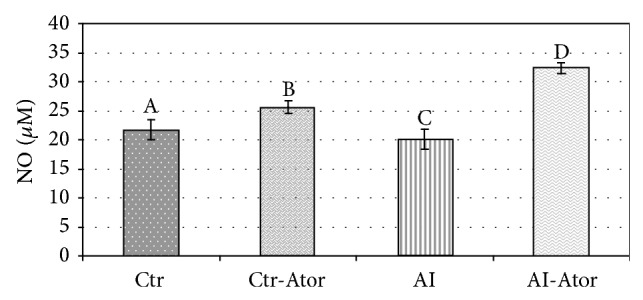
Behavior of plasmatic NO in rats with induced HF (*n* = 12). Crt versus Ctr.Ator ND; Ctr versus AI *P* < 0.01; Ctr versus AI-Ator *P* < 0.001; Ctr-Ator versus AI *P* < 0.001; Ctr-Ator versus AI-Ator *P* < 0.001; AI versus AI-Ator *P* < 0.001.

**Figure 3:**
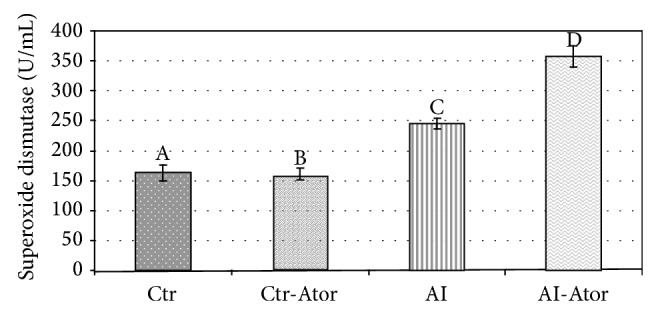
Enzymatic activity of SOD in rats with induced HF treated with atorvastatin (*n* = 12). Crt versus Ctr.Ator ND; Ctr versus AI *P* < 0.001; Ctr versus AI-Ator *P* < 0.001; Ctr-Ator versus AI *P* < 0.001; Ctr-Ator versus AI-Ator *P* < 0.001; AI versus AI-Ator *P* < 0.001.

The results of the plasmatic variables: fibrinogen, NO, and SOD activity, are shown in Figures 1, 2, and 3 for all groups.

The AI group showed higher fibrinogen values (291 ± 1.64) than the Ctr (202 ± 6.11) (*P* < 0.001). Compared to the AI group, the AI-Ator group treated for 75 days (187.70 ± 9.12) showed a significant decrease in fibrinogen (*P* < 0.001), but no significant difference was found between the groups Ctr-Ator (235.33 ± 3.45) and AI-Ator.

Compared to the Ctr (21.64 ± 1.73), the AI group (18.09 ± 3.96) revealed a significant decrease in NO bioavailability (*P* < 0.01). A marked increase in NO (*P* < 0.001) was found for AI-Ator (32.60 ± 6.01) compared to the Ctr group. Additionally the bioavailability of NO was almost the same for the Ctr-Ator (25.65 ± 2.53) and the Ctr group and these differences were not significant.

SOD activity was significantly increased in the AI group (245.42 ± 36.15) as compared to Ctr (163.33 ± 45.49) (*P* < 0.001). When compared to Ctr, a significant rise in enzymatic activity (*P* < 0.001) was observed in the AI-Ator group (357.75 ± 29.64). Comparison of the AI and treated AI-Ator groups revealed a significant difference between the two (*P* < 0.001). The differences between control group and the control group with treatment (158.30 ± 4.59) were not significant.

**Figure 4:**
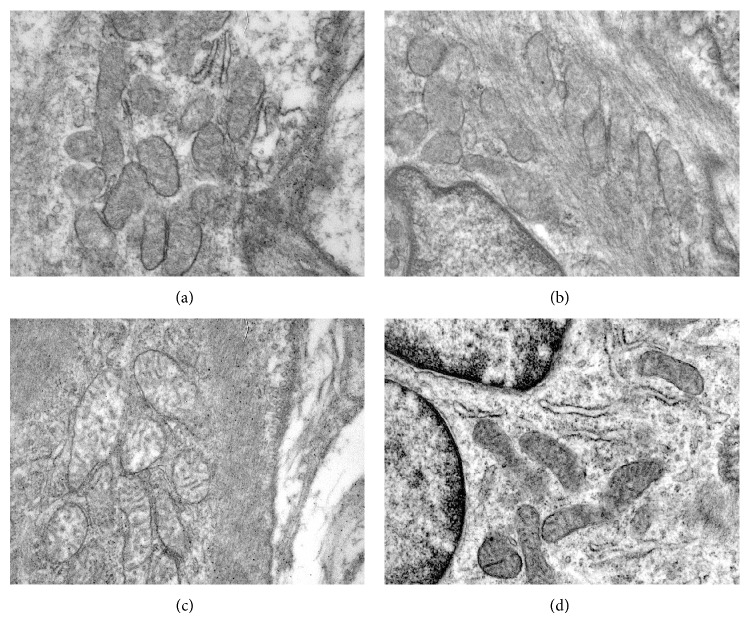
(a) Microphotograph of mitochondria in Ctr group, structure of membranes, and crests without changes and maintaining normal shape and size (arrow), 27800x; (b) Ctr + Ator group showing no changes in shape and size, 21560x; (c) AI group, showing an expansion of the intermembranous space, disorganization of crests, and turbid tumefaction (arrow), 27800x; (d) microphotograph of thoracic aorta of AI-Atorv group, showing mitochondria with unharmed membranes and normal mitochondrial crests, 21560x.

When the results of the groups with persistent hyperfibrinogenemia and decreased NO were analyzed, the total and mean number of mitochondria decreased significantly in the AI group (Figure 4) in comparison to the Ctr group (Figure 4) (*P* < 0.001). In addition, there was a marked expansion of the intermembrane space and clearance of the mitochondrial matrix, with disorganized crests and vacuoles and even an apparent absence of crests in some organelles. These changes are compatible with mitochondrial tumefaction. Furthermore, mitochondria presented perinuclear localization associated with several vesicles. Mitochondria in the AI-Ator group treated for 75 days (C) (Figure 4) revealed unharmed membranes and normal mitochondrial crests. Degree of alteration decreased from 3 to 18.75%, which was statistically significant in relation to untreated animals with induced HF (Table 1).

## 4. Discussion

In recent years, the study of the genesis of ischemic substrate vascular pathologies has incorporated the analysis and highlighted the importance of inflammatory components expressed by different biomarkers such as hyperfibrinogenemia. In the present study, HF was induced and increased by adrenal pathway which generated a downregulation process in relation to endogenous adrenaline, showing that the vascular lesions observed are produced by the proinflammatory and prooxidative action caused by HF and not by vascular adrenergic effects [4]. This has been shown in previous studies from our laboratory, along with the increase of TNF-*α* level in HF conditions, indicating the existence of endothelial activation [7].

Due to this endothelial homeostatic imbalance, there was a reduction in the bioavailability of NO in the AI group, suggesting that HF generates oxidative stress with a loss of regulation of vital functions in endothelial cells. The decrease in NO is considered the earliest phenomenon of endothelial dysfunction [15, 24]. Atorvastatin functions by directly binding to the regulatory site of *β*2 integrin, a protein involved in the inflammatory response since fibrinogen is part of it. When the expression of this protein is modulated, fibrinogen levels are reduced. Atorvastatin causes a reduction in the activation of platelets through different ways, decreasing the conversion of fibrinogen into fibrin. Fibrinogen increase is associated with the degree of endothelial dysfunction produced by these chemical mediators [25, 26]. It is likely that inducible NOS (iNOS) is activated, resulting in an increase in NO synthesis. However, NO does not reach its biological targets because its bioavailability is reduced as a result of reacting with O_2_^−^, whose concentrations are elevated by oxidative stress. This reaction is six times faster than that of superoxide anion with superoxide dismutase (SOD), leading to reduced bioavailability of NO, which is diverted to peroxynitrite formation, strong biological oxidant, additionally increasing oxidative stress [24].

Our results are consistent with those finding an increase in endothelial nitric oxide, the highly studied and documented pleiotropic effect of atorvastatin. This effect is produced by the action of the drug at several levels: inhibition of the expression, by the endothelial and smooth muscle cells and monocytes, of cytokines, chymosins, and growth factors which reduce eNOS enzyme expression, preventing endothelial cells from releasing an adequate level of NO [27]; reduction of free radical production, which favors the conversion of NO into peroxynitrites that are harmful to the cell; caveolin reduction and increase of eNOS enzyme activity [28]; and ARNm of eNOS stabilization through the signaling pathway of Rho/ROCK [29]. Furthermore, mevalonic acid can directly inhibit the NO synthesis in a process dependent on the inhibition of geranylgeranyl transferase [19, 25]. This behavior has an impact on mitochondria, generating morphological lesions such as those observed here, and alters the efficiency of respiratory chain coupling, increasing O_2_^−^ production, both typical effects of ischemic lesions. Due to the efficiency of the reaction of superoxide with NO, the local concentration of SOD is a determining factor of NO bioavailability. Increased SOD activity, which rises further with HF induction, comes from an adaptive response aimed at compensating oxidative stress that is manifested as an increase in the redox environment [18, 30]. The higher superoxide ion is the cellular signal that triggers the activation of extracellular SOD after obtaining its catalytic copper cofactor by increase of the Atox1 expression, a copper chaperon that activates extracellular SOD enzyme and positively regulates its transcription [31]. These changes in SOD activity are only expressed in subclinical atherosclerosis, as observed in the results. Our data suggest that relatively high SOD activity levels were not sufficient to compensate proatherogenic oxidative stress, maintain NO availability, and inhibit peroxynitrite formation. A vicious cycle of oxidative stress can cause atherogenesis [17, 28], leading to protein nitration and DNA and lipid damage, inducing an overexpression of redox genes, which would result in damages to the aortic layers.

During HF, we observed a reduction in the structure of mitochondrial crests and a condensation of the matrix, a condition called mitochondrial tumefaction. Matrix granules disappear and conserve a clear appearance; there is an increase of water in the matrix by alteration of cell membrane integrity, probably due to the transitory opening of the transition pores (TP) that open during ischemia and cause depolarization of the mitochondrial membrane potential and affect ion homeostasis, both in the cell as well as in the mitochondrion. This leads to matrix tumefaction and the subsequent breaking of the outer mitochondrial membrane [32]. Mitochondrial crests also disappeared (crystolysis), a lesion that is reversible during its initial stage, suggesting the need for an early pharmacological strategy. Although the mitochondrial number was not recovered, the increased enzymatic activity of this group allows us to infer that some mitochondrial biogenesis mechanisms have been set into motion, a process through which new mitochondria are formed from the transcription and translation of genes, both in the nuclear genome and in the mitochondrial genome. Both processes require the energy supplied by mitochondria since they play a central role in energy homeostasis, metabolism, and signaling; as a consequence, the abundance, morphology, and functional properties of mitochondria are corrected to satisfy specific energetic and metabolic demands [32]. The normalization of the mean mitochondrial number was achieved on day 75 when enzymatic activity was also tending towards normalization, indicating that once the mitochondrial number is recovered, enzymatic activity is also normalized. The beginning of mitochondrial biogenesis seems to be closely related to the effect that the mitochondrial number and enzymatic activity have on the recovery of eNOS activity, as shown by the increase in NO bioavailability, which is consistent with studies suggesting a close relationship between mitochondrial biogenesis and eNOS activity [32]. Both in human and animal models, the use of atorvastatin has been shown to reduce oxidative stress markers and improve atherogenic lesions in clinical doses, which could presumably be related to Rac inhibition [25, 33].

An increase in mitochondrial enzymatic activity could lead to a decrease in the production of mitochondrial ROS which depends on the mitochondrial metabolic state and is lower in metabolic states characterized by a high electron flow and a fast ATP synthesis, contributing to a reduction in oxidative stress [27]. On this basis, administration of atorvastatin could be a therapeutic option for the treatment of vascular disorders related to inflammation and oxidative stress, which is reflected in the improvement of biomarkers and a partial recovery of mitochondrial morphology.

## Figures and Tables

**Table 1:** Mitochondrial quantifications in the smooth muscle of the thoracic aorta of rats with atherogenesis induced by hyperfibrinogenemia and treated with atorvastatin.

Measurements	Ctr (A)	Ctr + Ator (B)	AI (C)	AI + Ator
Total number of mitochondria∗	54	53	37	41
Mean number of mitochondria∗	9 ± 0.30	8.8 ± 0.25	6.4 ± 0.97	7.5 ± 0.75
Mean area of mitochondria	465.61 ± 28.06 *μ*m^2^	458.88 ± 27.09 *μ*m^2^	792.068 ± 97.70 *μ*m^2^	667 ± 56.73 *μ*m^2^
Alteration				
Grade 1	87.25%	91.32%	5.4%	42.5%
Grade 2	12.75%	8.68%	24.32%	39.15%
Grade 3	0%	0%	70.27%	18.75%

^*^Total area measured 1986 *μ*m^2^. (*n* = 12 for all groups.)
